# Abstract of Proceedings of Buffalo Medical Association

**Published:** 1866-05

**Authors:** 


### ART. II.—*Abstract of Proceedings of Buffalo Medical Association.*

TUESDAY EVENING, March 5th, 1866.

The Association was called to order at 7½ o'clock by the President. Present—Drs. Ring, Gay, Cronyn, Strong, Eastman, Samo, Wetmore, Gleason, Little, Gould, Congar, Boardman and Johnson.

DR. JOHNSON moved that Dr. Abbott be invited to attend the meetings of this Association until he becomes eligible to membership. Carried.

DR. EASTMAN made a like motion in favor of Dr. H. S. Tafft, which was carried.

The minutes of the last meeting were read and adopted.

Voluntary communications being in order—

DR. GAY reported a case of diphtheria of unusual severity. The patient was a robust man of 26 years. On the first day of the disease all the usual general symptoms of the disease were present with no local disturbance until the second day, when he found a thick, firm exudate, complicated with abscess of the tonsil. The exudate came off and re-formed, dipping down into the larynx, making deglutition very difficult; most of the food consisting entirely of fluid, was returned through the nares. The abscess opened spontaneously, and healed two or three days after.

Treatment consisted of sol. subsulphate ferri xx, minims aqua mentha, syp. simplex āā ℥ss, in teaspoonful doses every 2 hours. Gave also chlorate potassa in sat. sol. Gave neither whisky or quinine. Believe the good results in this case due to the sol. subsulph. ferri, and that the remedies of greatest value in such cases are, first, the aqueous vapor; second, subsulphate ferri.

Dr. Strong remarked, that we are too apt to attribute the good effects of alcoholic stimulants to systemic affects. He believes that the local effects of alcoholic stimulants are valuable in diphtheria. Dr. S. related the case of a patient with diphtheria with but a small amount of exudate and constitutional symptoms well marked; pursued his usual plan of treatment until the fourth day, when he applied four leeches over the larynx with excellent effect, the patient convalescing rapidly. He also said that his experience had taught him that local depletion, and at the same time stimulants should be resorted to in many cases of inflammatory disease.

Dr. Eastman had not used sol. subsulph. ferri in diphtheria· Believes that aqueous vapor in many cases a very useful remedy. He related a case in which there was total loss of voice, used aqueous vapor and made application of ice over the larynx for twelve consecutive hours, when the patient was much better and recovered rapidly. Had also recently seen a case treated by another practitioner in which the patient seemed convalescing favorably, when edema of the glottis supervened, and death occurred in about ten minutes from the commencement of the edema.

Dr. Wetmore remarked that he employs the vapor from a saturated sol. of carbonate of soda, and thinks it superior to aqueous vapor. Often paints the membrane with this solution, and sometimes uses carb. soda in fine powder, directing the patient to inhale it. Has often seen neuralgia follow diphtheria, but thinks paralysis follows it very infrequently. Should hardly know how to treat diphtheria without iron, quinine and whisky.

Dr. Strong spoke favorably of aqueous vapor in croup, but remarked that he believed true croup almost always fatal.

Dr. Boardman reported a case of pleuro-pneumonia in a robust man, with pulse at 120, and short, painful respiration and slight cough and severe headache. Gave $\frac{1}{3}$ gr. morphia by hypodermic injection, and $\frac{1}{8}$ gr. morphia four hours after in conjunction with mustard poultice. Slept quite well through the night. In the morning found the pulse 100, and much less pain; headache gone. Treatment continued through the day. Sputa rusty and heavy. Next day found pulse 86; sputa lighter, but little pain; frebrile excitement nearly ceased. Patient convalesced rapidly. Believe the subcutaneous injection of the morphia very useful in this case.

Dr. Eastman said that the case related by Dr. Boardman was very interesting, and that the hypodermic use of morphia is many times very advantageous, and thinks its use in this manner will be of great value in the treatment of cholera and kindred affections.

Dr. Cronyn remarked that he had used morphia hypodermically in cholera with the best results. Dr. C. believes it the almost universal opinion that paralysis follows diphtheria. Has no faith in local treatment of this disease.

Dr. Boardman had found the hypodermic injection of morphia very useful in cholera-morbus. Had employed it in this manner in cases where nothing could be retained in the stomach, purging very violent. Soon after injection purging ceased. Gave small doses hyd. chl. mite, and the vomiting was also arrested. Thinks he should have lost the patient but for the injection.

Dr. Cronyn stated that in the case of the bullet-wound reported by him at a former meeting, the bullet is still in the boy's head. The boy is doing quite well. The right side of the face and the right leg is paralyzed. The right arm is atrophied.

Dr. Wetmore said, diphtheria is a general disease, sometimes with local disturbance and sometimes not. Sometimes with much exudation, or may be with much ulceration. If the exudation is taken off early its formation in the larynx is prevented.

Dr. Cronyn said that many more cases of diphtheria are reported than ever exist. The removal of the exudate will be of no more advantage than the removal of the skin. The disease is ameliorated only by such remedies as tend to eliminate the poison from the system. If the exudate will immediately re-form, of what use is it to remove it?

Report on prevailing diseases being in order, pneumonia, effemora, diarrhœa, variola and erysipelas were reported as prevailing.

Adjourned. T. M. Johnson, Sec'y.

---

### Annual Meeting, April 3d, 1866.

The Association was called to order at the usual hour by the President. Present—Drs. Lockwood, Ring, Samo, Gay, Wetmore, Trowbridge, Little, Cronyn, Tafft and Johnson.

The report of the last meeting was read and approved.

Dr. Cronyn presented to the Association George Beam, of Fort Erie, C. W., aged 14 years, who was five months ago accidentally shot with a pistol. The ball penetrated the frontal bone a line or two above the left supra-orbital ridge, and passed horizontally into the skull to the distance of about four inches. His right arm and leg are gradually recovering from the paralysis, and he seems likely to recover entirely.

The annual report of the treasurer was read and referred to the board of auditors.

On motion of Dr. Gay the subscription of Dr. J. S. Trowbridge was canceled.

Election of officers being in order the following gentlemen were elected for the ensuing year:

| | |
|---|---|
| President, - - | Dr. William Gould. |
| Vice President, - - | " J. S. Trowbridge. |
| Secretary, - - | " T. M. Johnson. |
| Treasurer, - - - | " T. T. Lockwood. |
| Librarian, - - | " J. B. Samo. |

On motion of Dr. Trowbidge, the Society was requested to tender the use of the rooms of the Association, to the American Society for the Advancement of Science, during the meeting of that Society in August next.

Dr. Lockwood presented a specimen of the bark and also the root of the indigenous black ash, and would call the attention of the Association to the subject of our indiginous materia medica. He stated that the bark should be procured when it contains the sap. It may be given in decoction or extract, and is a valuable stimulant, tonic and antiperiodic. It is quite extensively used in the country as a vermifuge. Dr. L. has used it quite extensively in intermittent fever with good results, and thinks it very efficient after the paroxysms have been controlled by quinine.

Dr. Wetmore moved that Drs. A. Kaffenburgh and Thomas Lothrop be invited to seats in the Association until they can complete their membership. Carried. Adjourned.

T. M. Johnson, Sec'y.

*Action taken by Physicians in Buffalo in view of the probable appearance of Cholera.*

Pursuant to a resolution of the Buffalo Medical Association passed at the regular meeting of February 6th, a meeting of the regular profession of the city and vicinity was held at the rooms of the Buffalo Medical Association on the 13th of February, and Dr. Josiah Barnes was elected Chairman of the meeting, and Dr. T. M. Johnson elected Secretary.

Dr. Rochester stated that the object of the meeting was to consult together upon the best means for the protection of our citizens in case cholera should prevail here during the ensuing summer. He read a circular from the Board of Hygiene and Public Health of New York City, recommending that such action be taken by the physicians of Buffalo as will result in immediate and efficient sanitary measures.

On motion of Dr. Rochester the Chairman was directed to appoint a committee of five physicians to wait on the Mayor and Board of Health and tender coöperation with them in devising and carrying out such sanitary measures as may seem advisable.

After considerable discussion, in which there was general participation, the motion was carried. The Chairman appointed Drs. Rochester, Gay, J. R. Lothrop, Wyckoff and Haunstein, said Committee.

On motion of Dr. C. W. Harvey the Committee was directed to take such course as was necessary to fully carry out the desired objects and report at an early day.

In obedience to this motion the Committee have issued to the Board of Health, and to the citizens the following

CIRCULAR.

Inasmuch as cholera will probably visit this city, and as much may be done to prevent or mitigate its prevalence, the following is issued for the information of our citizens, and to disarm them of groundless and unnecessary fear:

There is no epidemic of equal severity, so entirely under judicious hygienic control. This refers to cleanliness of person and premises, the avoidance of crowded and ill-ventilated apartments, and especially to the avoidance of excesses in eating and drinking. Cholera is always more prevalent in warm and moist than in warm and dry weather. There is an affinity between it and dampness; hence nothing is more prejudicial than watering floors, walks or streets at nightfall, under the mistaken idea that by thus cooling the ground, a more healthful atmosphere is secured. Cholera is almost invariably preceded by diarrhœa from one to four days. This is, in fact, its first and *curable* stage, but because the diarrhœa is usually painless, it is neglected or trifled with. No one, with even a very slight diarrhœa, (cholera prevailing) should attempt to pursue his ordinary avocations. He should go to bed, or at least maintain a recumbent position, should be covered up warm, and should take no food, or but little, and that very light and digestible. Should the diarrhœa continue, he should *at once* consult his physician.

Nearly every one may have, through his medical adviser, some simple preparation which will check or arrest the diarrhœa until the physician's personal attendance can be secured. Nostrums, either to cure or to prevent should not be taken. Cathartics of every description should be avoided. If cholera is communicable, it is probably so through the stools, hence all evacuations of diarrhœa or cholera should be at once removed from within doors, and privies and water-closets should be kept sedulously clean and covered. A cheap and effectual disinfectant is found in copperas, (sulphate of iron.) Of this, one pound dissolved in a large pail of water, may be emptied occasionally in out-houses, drains and cesspools. No dirty water should be allowed to stand in tubs or slop-pails; nor, if possible, in streets or gutters. No excavation of soil should be made, and no privies, drains, or old dirt-heaps should be disturbed in warm weather. All this should be done prior to May. Many persons, in cholera epidemics, use ardent spirits more or less freely as a supposed preventive. This is a great error. Drunkards and intemperate persons are much more apt to be attacked than those of contrary habits. It has always been observed, that independently of the influence of atmospheric conditions, cholera has notably been more prevalent on Sunday nights and on Mondays than on any other nights and days of the week. This is undoubtedly rightly ascribed to the intemperance and dissipation in which many are in the habit of indulging on Sundays.

Another error into which many are apt to fall is, to place themselves upon a restricted diet. This is believed to be, not only unnecessary, but injudicious. Plain, substantial, well-cooked meals, in which fresh animal and vegetable food both enter, are better than a dietary of bread, rice and salted meats. Fresh and ripe fruits and vegetables in their season, are healthful, but the same when crude, wilted, or fermented, are at all times pernicious.

The above statement, it is believed, covers the material points pertaining to the prevention and mitigation of epidemic cholera, and is respectfully submitted.

THOS. F. ROCHESTER,
C. C. F. GAY,
JOHN HAUNSTEIN,
C. C. WYCKOFF,
J. R. LOTHROP.

---

*To the Board of Health of the City of Buffalo:*

It is respectfully suggested to your honorable body, that, in order to increase the efficiency of your sanitary measures, that the city be divided into districts, and that ten Medical Inspectors be appointed—one for each district—who, in case of the approach or invasion of cholera, shall engage and be ready to serve, subject to your orders.

It shall be the duty of these Inspectors, to make house to house visitations, examine the premises thoroughly, and give such hygienic and special directions as may be necessary, prescribing and furnishing medicine *gratuitously* in cases of destitution and urgency.

It shall be their duty to devote at least five hours daily to this work, and to report in writing to the Board of Health daily, the extent and result of their investigations.

Each Medical Inspector shall receive five dollars per diem for such period as the Board of Health shall direct them to continue their labors.

THOS. F. ROCHESTER,
C. C. F. GAY,
JOHN HAUNSTEIN,
C. C. WYCKOFF,
J. R. LOTHROP.

